# Development and validation of a mental health screening tool for asylum-seekers and refugees: the STAR-MH

**DOI:** 10.1186/s12888-018-1660-8

**Published:** 2018-03-16

**Authors:** Debbie C. Hocking, Serafino G. Mancuso, Suresh Sundram

**Affiliations:** 1 Cabrini Institute, 154 Wattletree Road, Malvern, VIC 3144 Australia; 2 Florey Institute of Neuroscience and Mental Health, 30 Royal Parade (Cnr Genetics Lane), Parkville, VIC 3052 Australia; 3 Department of Psychiatry, Faculty of Medicine, Dentistry and Health Sciences, University of Melbourne, Parkville, VIC 3010 Australia; 4 Department of Psychiatry, School of Clinical Sciences, Monash University, Clayton, VIC 3168 Australia; 5 Adult Psychiatry, Monash Medical Centre, Clayton, VIC 3168 Australia

**Keywords:** Asylum-seekers, Refugees, Mental health screening, Tool development, Post-traumatic stress disorder, Depression

## Abstract

**Background:**

There is no screening tool for major depressive disorder (MDD) or post-traumatic stress disorder (PTSD) in asylum-seekers or refugees (ASR) that can be readily administered by non-mental health workers. Hence, we aimed to develop a brief, sensitive and rapidly administrable tool for non-mental health workers to screen for MDD and PTSD in ASR.

**Methods:**

The screening tool was developed from an extant dataset (*n* = 121) of multiply screened ASR and tested prospectively (*N* = 192) against the M.I.N.I. (Mini International Neuropsychiatric Interview) structured psychiatric interview. Rasch, Differential Item Functioning and ROC analyses evaluated the psychometric properties and tool utility.

**Results:**

A 9-item tool with a median administration time of six minutes was generated, comprising two ‘immediate screen-in’ items, and a 7-item scale. The prevalence of PTSD &/or MDD using the M.I.N.I. was 32%, whilst 99% of other diagnosed mental disorders were comorbid with one or both of these. Using a cut-score of ≥2, the tool provided a sensitivity of 0.93, specificity of 0.75 and predictive accuracy of 80.7%.

**Conclusions:**

A brief sensitive screening tool with robust psychometric properties that was easy to administer at the agency of first presentation was developed to facilitate mental health referrals for asylum-seekers and new refugees.

**Electronic supplementary material:**

The online version of this article (10.1186/s12888-018-1660-8) contains supplementary material, which is available to authorized users.

## Background

The world currently has the largest number of displaced persons at any time in history [1]. This has seen increasing numbers of forced migrants entering industrialised countries [1], intensifying the challenge to efficiently screen health conditions. In most countries asylum-seekers are not screened for mental health problems at any point during the asylum process [2], which is similarly the case for a large proportion of newly resettled refugees [3–7].

Compounding this is the knowledge that forced migrant populations have high rates of mental disorders, with major depressive disorder (MDD) and post-traumatic stress disorder (PTSD) in particular being many fold higher than in host [8] and non-forced migrant populations [9]. A large meta-analysis of refugees and other conflict-affected persons reported adjusted weighted prevalence rates of 30% for MDD and PTSD [10], suggesting these to be the most widespread mental disorders in this population with even higher rates reported in asylum-seekers [11–14].

The burgeoning number of displaced persons globally and their disproportionately high rates of mental disorders have prompted the World Health Organisation (WHO) to call upon treatment services to be responsive to the needs of asylum-seekers and refugees [15]. Yet, utilisation of mental health services is comparatively low in this population [16] for reasons including lack of accessibility, poverty, poor language comprehension, lack of knowledge of services, cultural factors and stigma [4]. Furthermore, increasingly time-constrained agencies have to contend with increasing need whilst grappling with limited human resources. Several of these issues could be addressed by the availability of a brief, sensitive and rapidly administrable screening tool by non-mental health workers which would facilitate triaging of asylum seekers and refugees with mental health problems to be referred to an appropriate health service.

Extant mental health screening tools do not fully meet these aforementioned criteria with several well-utilised tools having a number of drawbacks. These include not being validated in forced migrant populations (e.g., K10, K6, BAI, DASS-21, PTSD-8, GHQ-12) [17, 18]; too prolonged to facilitate rapid screening of large populations (e.g., DASS-21, RHS-15; HSCL-25; HTQ); screening for distress rather than disorder and lacking predictive validity against a standardised psychiatric interview (e.g., K10; RHS-15; WHO-5; SRQ-20) [19]; or screening for either MDD or PTSD – not both [20, 21].

Despite being one of the more commonly used screening tools for depression and anxiety, a recent review raised concerns about the lack of evidence for the validity and cultural equivalence of the K10, including variation between ethnic/linguistic groups for studies with multicultural samples [17]. The SRQ-20 was developed to screen for psychiatric disturbance, but primarily for those in developing countries, and has not established its predictive validity against a standardised psychiatric interview [19]. Whilst the RHS-15 [3] was developed for refugee populations, it was designed to be administered in clinical settings, and has not been validated in asylum-seeker populations or against an acceptable gold standard [3].

A high proportion of mental disorders in the general population go undetected by healthcare professionals in the course of their routine work [22–24] and it would be predicted that non-mental health trained workers would be even less likely to identify possible mental disorders in their forced migrant clients. Consequently, we strove to develop a screening instrument that could be utilised by non-mental health workers across a variety of contexts, to minimise administrator-burden whilst increasing the likelihood of client uptake. To maximise the utility of such an instrument, it also would need to have efficacy in linguistically diverse and potentially under-resourced settings, using interpreters rather than undertaking multiple translations.

The present paper reports on the development and validation of a brief screening tool for PTSD and MDD in adult asylum-seekers and refugees (STAR-MH) that is suitable for use by non-mental health workers.

## Methods

### Procedure

**Fig. 1:**
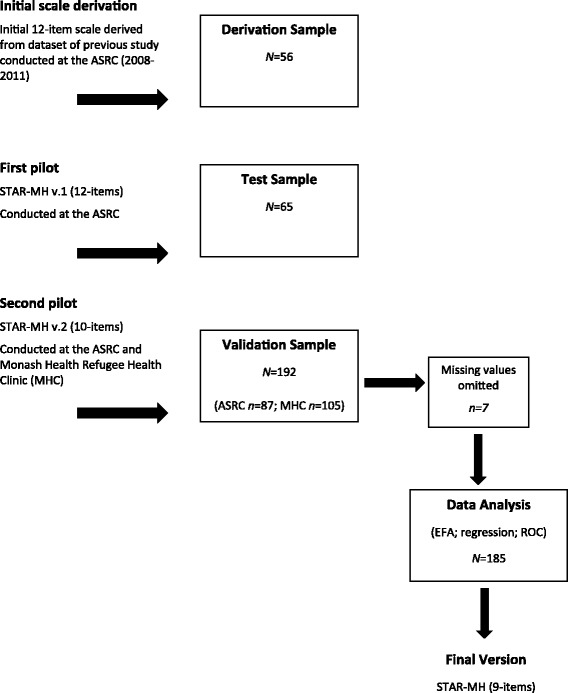
Sampling and iterative development of the STAR-MH

A visual overview of the data sampling process is presented in Fig. 1 which comprise a derivation sample – from which the initial 12-items scale was derived, and two pilot samples (for the initial 12-item version, and subsequent 10-item versions). The final version was a 9-item scale, which forms the basis of the Results section.

All administrators of the STAR-MH were briefed prior to each pilot study on the importance of adhering to the research protocol (such as delivering each item neutrally, without elaboration; providing written feedback on the process) and instructed about risk management processes should the need arise. Administrators were accustomed to working with interpreters. The tool itself was designed to be sufficiently simple to not require specific training to administer.

Given the purpose was to develop a mental health screening tool for forced migrants, participants were unselected by country of origin. Hence, the tool was not translated, but instead utilised in situ translation by professional telephone or face-to-face interpreters in the language required by participants as they consecutively presented to the recruiting agency.

The STAR-MH items were derived from scales considered to be gold standard instruments for measuring symptoms of depression, anxiety and PTSD in individuals of refugee-like background.

Community leaders from the cultural and linguistic communities with the greatest numbers of forced migrants in Australia (Sri Lankan/Tamil; Iranian/Farsi and Afghan/Dari) were consulted to ascertain the cultural appropriateness and utility of the tool.

### Derivation sample

The initial 12-item version of the STAR-MH [25], was derived from a sample of 56 ASR from a previous study [14] whom had completed four self-report questionnaires (Harvard Trauma Questionnaire-R, Parts I – trauma experiences & IV – PTSD and refugee-specific symptoms, and Hopkins Symptom Checklist-25 [26]; Post-Migration Living Difficulties Checklist [27]; and Psychiatric Epidemiology Research Interview—Demoralization Scale [28]) and the M.I.N.I. (Mini International Neuropsychiatric Interview) [29]. Scaled items from the four questionnaires were dichotomised, with values ≥3 designating clinical relevance. All 153 items were then entered into chi-square analyses to establish the sensitivity (SN) and specificity (SP) of each item against M.I.N.I diagnoses of PTSD and MDD. Only statistically significant items (Kappa statistic) were retained, resulting in 66 items.

Spearman’s Rho correlations were then performed between the 66 items and PTSD and MDD diagnoses. All items with a correlation ≥0.7 and a predictive accuracy of ≥0.85 for PTSD and/or MDD were retained and after two items were discarded due to redundancy nine items remained. Three ‘immediate screen-in’ items were included on clinical grounds, resulting in an initial 12-item version of the screening tool. A quantitative and qualitative administrator feedback section was also included to inform the second iteration of the tool (data not shown).

### Test sample

The initial 12-item version was evaluated at a community based asylum seeker welfare centre (Asylum Seeker Resource Centre, ASRC), with a consecutive sample of asylum-seekers (*N* = 65) being recruited through the (non-health) casework program. The sample was inclusive of adults (≥18 years) recently engaged with the ASRC (≤ 6 months) who had not been diagnosed with or treated for a psychiatric disorder since arriving in Australia. Eight ASRC casework program volunteers from a range of non-mental health backgrounds, such as university students, administration, nurses and general practitioners (GP) were briefed on the research protocol for the tool. All screened participants were subsequently administered the M.I.N.I by a researcher (DH), who was blind to the screening results. During the validation interview, demographic information was collected and participants were administered the HTQ-R (Part IV) and HSCL-25. Interpreters were utilised as necessary for both the screening and the validation interview.

Receiver operating characteristic (ROC) curve analyses were conducted on the 12 items with the test sample, resulting in five items being retained.

Given the objective was to identify likely caseness of either MDD or PTSD and because of the substantial comorbidity, both diagnoses were treated as a single outcome variable for the chi-square analyses below.

The statistical power was increased by pooling the derivation and test sample data. Hence, all 65 HTQ-R and HSCL-25 items were entered into chi-square analyses (*N* = 121) to ascertain the SN and SP of each item against M.I.N.I diagnoses of PTSD and MDD. Items with ≥85% SN and ≥ 75% SP were retained, resulting in 10 items in addition to those retained from the ROC analysis. Classification and Regression Tree (CART) analyses were then conducted for these 15 items with the total dataset (*N* = 121). This resulted in eight items being retained in addition to two of the original three ‘immediate screen in items’ from the chi-square analyses. The third ‘immediate screen in’ item was discarded due to poor predictive accuracy.

The resulting second iteration was a 10-item STAR-MH, the items of which are presented in Table 1, including the scales from which the items were derived.

**Table 1:** Screening tool for asylum-seeker and refugee mental health (STAR-MH) items

	STAR-MH Item	Scale derivation
1.^a^	Have you ever seen a doctor or health worker, gone to hospital, or taken medicines for your ‘nerves’? (i.e. *mental or emotional health*)	
2.^a^	Have you ***often*** wished you were dead, wanted to kill yourself or ever attempted suicide or harmed yourself?	
3.^b^	Have you felt ***very*** restless, like you can’t keep still?	HSCL-10 (anxiety item)
4.	Have you lost interest in things? (i.e. *things you usually enjoy*)	HSCL-23 (depression item)
5.	Have you worried about going crazy or ‘losing your mind’?	PERI-D-11 (also HTQ-24 ^d^; trauma item)
6.^c^	Have you had ***a lot*** of trouble sleeping?	HSCL-16 & HTQ-8 (depression and trauma item)
7.	Have you felt ***very*** fearful? (i.e. *scared or afraid*)	HSCL-2 (anxiety item)
8.	Have you felt ***very*** trapped or caught? (e.g. *like you are trapped in a situation you cannot get out of*)	HSCL-21 (depression item)
9.	Have you had ***a lot*** of pain in your body?	HTQ-20 (refugee-specific trauma item)
10.	Have you felt ***very*** worthless? (i.e. *like you have no worth or value*)	HSCL-25 (depression item)

^a^Immediate screen-in item

^b^Items 3–10 timeframe is the last 4 weeks

^c^Excluded from final 9-item scale

^d^Item from the original HTQ (Original Version)

### Validation sample

The 10-item screening tool was piloted with consecutive sample populations of ASR at two sites in Victoria, Australia: the ASRC (*n =* 87), and Monash Health Community (MHC) (i.e., Refugee Health Clinic; Monash Health Dental Clinic) (*n =* 105). In addition, a representative sample of administrators was enlisted to test the external validity of the tool.

ASRC participants were recruited through the casework and health programs. The recruitment strategy from the casework program involved screening tool administrators contacting all ASRC ‘general access-listed’ clients to ascertain eligibility and conduct face-to-face screening with consenting individuals. Consecutive ‘walk in’ patients presenting to the health program (non-mental health) who were eligible and consented were variously screened by a nurse, GP or untrained ASRC volunteer. Similarly, eligible consecutive patients presenting to the MHC were screened by a bicultural worker, nurse, GP or interpreter. All administrators were briefed on the research protocol for the tool and interpreters were utilised as required.

All screened participants were subsequently administered the M.I.N.I by a research team member (DH), who was blind to all screening results, using interpreters as required.

### Instruments

#### The mini international neuropsychiatric interview (M.I.N.I. 6.0)

The Mini International Neuropsychiatric Interview 6.0 (M.I.N.I) [29] is a brief, structured psychiatric interview developed in the United States and Europe for assessing the presence of DSM-IV and ICD-10 psychiatric disorders. It has been found to have sound SN (i.e., ≥ 0.70 for all but three of the modules), SP, negative predictive values and efficiency (i.e., ≥ of 0.85 across all diagnoses) when measured against the SCID [30]. Additionally, the majority of kappa values have been reported above 0.75, indicating good test-retest reliability, with inter-rater reliability also found to be high when validated with the SCID (i.e., 0.79–1.00) [30].

The M.I.N.I was chosen as the diagnostic instrument by which to validate the STAR-MH due to its brevity of administration compared to the SCID and CIDI [30, 31]; and its application within forced migrant populations [2, 32–34]. All MINI modules were applied, with the exception of Antisocial Personality disorder and Anorexia and Bulimia Nervosa due to i) focusing on prevalence of mental illness and ii) eating disorders being exceedingly uncommon in adult refugee populations.

#### Self-report measures

Items from the above measures (Harvard Trauma Questionnaire-R; Hopkins Symptom Checklist-25; Post-Migration Living Difficulties Checklist; and Psychiatric Epidemiology Research Interview—Demoralization Scale) contributed a pool of responses for potential inclusion in iterations of the STAR-MH.

#### Hopkins symptom Checklist-25 (HSCL-25)

The HSCL-25 [26] is divided into two parts: anxiety symptoms (Part I, 10 items, questions 1–10) and depression symptoms (Part II, 15 items, questions 11–25), with the Total Scale measuring ‘nonspecific emotional distress’. All items are coded 1 *(not at all)*, 2 *(a little)*, 3 *(quite a bit)* and 4 *(extremely)* indicating the degree of distress within the previous seven days.

The HSCL-25 has been translated into several languages [26] and used in many studies with forced migrant populations [35]. It was one of only two instruments adapted for refugee populations (the other was the Beck Depression Inventory) which met all five criteria (i.e., Purpose, Construct definition, Design, Developmental process, Reliability and validity) in a critical review of the validity and reliability of psychometric tools to measure mental health status in forced migrants [35]. Demonstrating very good predictive validity for diagnosed depression (SN = 0.88; SP = 0.73) [35], empirical studies have determined the depression items to be consistent with the DSM-IV diagnosis of major depression [26]. Furthermore, the HSCL-25 was found to have high SN (0.93) and SP (0.76) in detecting the presence of any major DSM-III-R-defined Axis I disorder in three Indochinese populations [36].

The HSCL-25 has demonstrated sound reliability in clinical refugee samples [35, 37, 38], having exhibited excellent test-retest reliability (*r* = 0.89) and internal consistency, which has been found to exceed 0.88 in refugee samples [37, 39].

#### Harvard trauma questionnaire – Revised (HTQ-R)

The Harvard Trauma Questionnaire (HTQ) [26] was designed to assess trauma related to mass violence and its sequelae, and has been used in numerous studies with forced migrant populations. It has been validated in several non-Western populations (e.g., Cambodian, Japanese, Vietnamese, Lao, Bosnian and Croatian) and met four of five criteria in a critical evaluation of instruments used to measure refugee trauma and health status [35].

The HTQ-R comprises four parts: Part 1: trauma events; Part 2: personal description; Part 3: head injury; Part 4: trauma symptoms. Only parts I and IV were included in the protocol, to assess previous traumatic events and PTSD symptoms, respectively, as both are established predictors of PTSD and are associated with other mental disorders, such as depression. Part IV comprises 40 items of trauma symptoms using a scale of 1 *(not at all)*, 2 *(a little)*, 3 *(quite a bit)*, and 4 *(extremely)* indicating the degree of distress within the previous seven days. The first 16 items (PTSD subscale) were derived from the DSM-IV criteria for post-traumatic stress disorder [26]. The remaining 24 items constitute a ‘refugee-specific’ subscale which measure self-perceived level of functioning and social disability, and which may be more highly correlated with trauma-related distress than the symptoms of PTSD [26].

The HTQ has demonstrated excellent statistical properties, including high interrater reliability (K = 0.93), scale test-retest reliability (1 week*, r* = 0.89); and internal scale consistency (α = 0.90) for the traumatic events sale (Part I). The trauma symptoms scale (Part IV) has demonstrated high interrater reliability (K = 0.98), scale test-retest reliability (1 week, *r* = 0.92); and internal scale consistency (α = 0.96). The PTSD items (Part IV, 1–16) have exhibited reasonable SN (0.78) and SP (0.65) as a screening instrument for PTSD, however, the additional ‘refugee specific’ items (Part IV) increased the SP to 0.78 (SN remained unchanged).

Both the HSCL-25 and HTQ are considered to be gold standard self-report measures of psychiatric symptomatology in forced migrant populations, having demonstrated robust psychometric properties [26, 35], and are among the most widely used self-report measures for psychological distress in forced migrants.

#### Psychiatric epidemiology research interview–demoralisation scale (PERI-D)

The PERI-D [28] demoralisation scale comprises 27 items which measure nonspecific distress using a five-point scale ranging from 0 (‘never’) to 4 (‘very often’) with a composite score calculated by dividing the total score by the number of items completed. It has been employed in a conflict-affected population [40], clinical [41] and community [42] populations, and with Jewish and Middle Eastern immigrants [43–46].

#### Post-migration living difficulties checklist (PMLDC)

The PMLDC [47] is a 23-item checklist to assess current life stressors of asylum-seekers, having been developed from an ad hoc checklist of a range of typical problems reported by asylum-seekers [48]. Hence, it is an important instrument to measure life experiences other than war [35]. Each item is rated on a 5-point scale from ‘no problem’ to ‘very serious problem’, with a composite score determined. The PMLDC has been used [47, 49, 50] or adapted for use [51, 52] in forced migrant populations internationally.

### Statistical analysis

All data were analysed using *R* version 3.2.2 [53]. The *eRm* [54] and *ltm* [55] packages were used to conduct the Rasch modelling.

### Rasch analysis

Rasch analysis was conducted to examine the construct validity of the STAR-MH at instrument, person, and item levels. Rasch modelling is a probabilistic approach to estimate the difficulty of questionnaire items, which assumes that a single latent construct accounts for item responses [56]. The probability of a person endorsing an item is a logistic function of the item difficulty and person ability [57]. This logistic function is an interval scale with a midpoint of 0. The items are ordered on the scale in descending order according to their difficulty level. Items on the top of the scale are considered more difficult and have lower probabilities that a person endorses it, whereas items at the bottom of the scale are deemed less difficult and have a high probability of being endorsed [57]. In the present context, the latent variable is psychological distress and a high item score indicates higher levels of psychological distress. Therefore, the interpretation of item difficulty is such that a high item difficulty estimate relates to fewer people endorsing the symptom of psychological distress. Conversely, individuals achieving a lower score on the STAR-MH items experience lower psychological distress and are assigned a lower person ability.

#### Dimensionality and local item dependence

The Rasch model assumes that a set of items are unidimensional, with items being locally independent from each other. The procedure of Drasgow and Lissak [58] was used to check the dimensionality of the dichotomously scored STAR-MH responses using modified parallel analysis with 2000 Monte Carlo samples. The test is implemented in the *ltm* [55] package and a non-significant *p*-value is indicative of unidimensionality.

Local dependency detection was conducted by using Ponocny’s “T1” test [59] for local dependence assessing increased inter-item correlations for all 21 possible item pair combinations. This test is implemented by the *NPtest* function in the *eRm* package [54] and a statistically significant test at *p* < .05 is indicative of local dependence.

#### Item fit

The information-weighted fit (*infit*) and the outlier-sensitive fit (*outfit*) were used to test whether the items fit the expected model. The infit statistic is more sensitive to unexpected responses to items closest to the person’s ability level, whereas outfit statistic is more sensitive to unexpected responses to items further away from the person’s ability [60]. Items with respective infit and outfit values between 0.60 and 1.40 are considered a good fit to the Rasch model [61]. In addition, standardized infit (*infit-t*) and outfit (*outfit-t*) statistics and an associated chi-squared statistic were calculated. Items with standardized infit and outfit values of between − 2.50 and 2.50 are deemed to indicate adequate fit to the model. To account for multiple testing using Bonferroni corrections, the chi-squared *p*-value was multiplied by the number of items in the Rasch model. For this series of analyses, an adjusted *p*-value of less than 0.05 was considered statistically significant.

#### Differential item functioning (DIF)

DIF or item bias can occur when different groups within a sample, despite having the same levels of the latent trait (i.e., psychological distress), respond in a different manner to an individual item [57, 62]. There should be no differences in the probability to endorse a certain item based on the subgroups. The logistic regression method [63] implemented by the *difLogistic* function in the *difR* package [64] was used to assess DIF for subgroups based on sex, age, interpreter use, support agency, country of origin, marital status, travel mode, and post-migration detention status. Age was dichotomised based on the median age (18–33 vs. 34+), while country of origin was dichotomised into Southern Asia or other. An effect size based on Nagelkerke’s *R*^2^ statistic [65] provides a quantification of DIF [66]. The effect sizes are classified as “negligible” (*R*^2^ < 0.035), “moderate” (0.035 ≤ *R*^2^ ≤ 0.07), “large” (*R*^2^ > 0.07) [67]. Given the multiple comparisons for each item, the Benjamini and Hochberg (BH) false discovery rate was applied to control for Type I error. This is the recommended adjustment when assessing DIF using logistic regression [68].

#### Internal consistency

The Person Separation Index (PSI) provides an indication of the internal consistency of the scale and is interpreted in the same manner as the Cronbach alpha coefficient [62]. While a PSI of 0.70 is considered a minimal value for group or research use and 0.85 for individual or clinical use [69], it can be influenced by the number of items in the scale. For scales with few items, it is recommended to report the mean inter-item correlation, with an optimal range of between 0.20 and 0.40 [70].

### Receive operating characteristic (ROC) analyses

A receiver operating characteristics (ROC) plot was used to assess the sensitivity and specificity of the STAR-MH in discriminating between participants with caseness for PTSD/MDD and those without. Sensitivity is the proportion of true positives that is correctly identified by the test, while specificity is the proportion of true negatives that is correctly identified by the test [71]. A ROC plot is obtained by calculating the sensitivity and specificity of every observed data value and plotting sensitivity against 1 – specificity. The area under the ROC curve (AUC) is the most used measure of the accuracy of a diagnostic test and ranges between 0.5 and 1, with 0.5 indicating poor accuracy and 1 representing perfect accuracy. Furthermore, a ROC analysis is independent of disease prevalence [71].

The bootstrapped optimism-corrected AUC was calculated to estimate the deterioration that the model will have when applied to new participants using the algorithm of Harrell et al. [72]. This approach outperforms split-sample validation, particularly when the sample size is limited [72, 73]. If the bootstrap optimism-corrected AUC shows acceptable predictive accuracy, then the model is validated [72]. As recommended by Harrell et al. [72], 200 resamples with replacement were drawn from the original data (*N* = 185).

Youden’s *J* statistic [74] was used to determine the optimal cut-off score for the STAR-MH. This was calculated as the sum of sensitivity and specificity for each cut-off value to indicate the test score at which the greatest proportion of individuals is correctly identified as being cases and non-cases. The positive and negative likelihood ratios (PLR/NLR), positive and negative predictive values (PPV/NPV), and predictive accuracy were also calculated for each cut-off score. The *R* package *pROC* [75] was used to conduct the ROC analyses.

## Results

The final pilot (‘validation sample’) screened and psychiatrically evaluated 192 participants from 36 countries and 27 different language groups. Eighty-seven participants were recruited through the ASRC, whilst 85 declined, and 105 individuals were recruited through the MHC whilst 10 declined. Overall, this represents a participation rate of 66.9%.

The STAR-MH was deemed to be culturally appropriate by community leaders of the three largest language groups represented in the sample (i.e., Farsi, Dari and Tamil, comprising 43% of the sample). All confirmed the cultural validity of the tool and believed the tool would be a useful resource in their respective communities.

Participants ranged from 19 to 82 years, with a median age of 33 years (IQR 36–43), and median time in Australia of 2 years (IQR 0.70–3.11). Demographic and clinical characteristics of the sample (*N* = 192) are presented in Additional file 1: Table S1.

Twenty-eight non-mental health workers administered the screening tool, with a median administration time of six minutes (*IQR =* 5–7), irrespective of whether an interpreter was used (i.e., *Md* = 5, *IQR* = 4–7 without interpreter; *Md* = 6, *IQR* = 5–7 with interpreter). The M.I.N.I validation interview took place a median of 5.50 (IQR 0–9) days post-screen and identified rates of MDD and PTSD at 29.7% and 19.9% respectively, with the prevalence of PTSD and/or MDD being 32.3%. Sixty-four participants (33.3%) met criteria for at least one mental disorder and there were only two cases (both of whom were diagnosed with substance use disorder [SUD]; one was nil current) that were not comorbid with PTSD or MDD. Hence for 99% of the total sample, other diagnosed mental disorders (i.e., GAD 3%; panic disorder 2%; SUD 1.5%; psychosis 1%; OCD 0.5%; agoraphobia 0.5%) were comorbid with PTSD or MDD. Suicidality was 6.8%.

### Preliminary analyses

Cases with missing responses (*N* = 7, 3.6%) to the STAR-MH were omitted from further analyses. Thus, the resulting sample size was 185 participants (Table 2). Table 3 presents the response frequencies for the eight items which comprised the prospectively tested scale (see *Validation Sample*), excluding the two immediate screen-in items. Item 9 had two missing values and item 10 had five. The response frequencies for the eight items for the total sample (*N* = 192) can be found in Additional file 2: Table S2.

**Table 2:** Demographic and clinical variables of participants (*N* = 185)

	*N* ^a^ (%) ^b^
Gender	
Male	129 (69.7)
Age group	
18–24	24 (13.0)
25–34	75 (40.5)
35–44	48 (25.9)
45–54	27 (14.6)
55+	11 (5.9)
Marital status	
Partnered	111 (60.0)
Mode of Arrival	
Irregular maritime arrival	98 (53.0)
Continent of Origin (UN geoscheme)	
Africa	
East Africa	17 (9.2)
North Africa	10 (5.4)
West Africa	6 (3.2)
Asia	
South Asia	98 (53.0)
South-East Asia	37 (20.0)
West Asia	8 (4.3)
Other	9 (4.9)
Interpreter required	
Yes (screening)	113 (61.1)
Yes (interview)	119 (64.3)
Pre-migration camp/detention	
Yes	26 (14.3)
Post-migration Immigration Detention	
Yes	99 (53.5)
Years in Australia	
< 1 year	34 (18.6)
1–2 years	34 (18.6)
2–3 years	75 (41.0)
> 3 years	40 (21.9)
Residency status	
Temporary Visa (Asylum-seeker)	187 (97.3)
Permanent Residency (Refugee)	5 (2.7)
Mental health diagnosis (Australia)	
Yes	8 (4.6)
MINI-assessed Post-traumatic stress disorder (PTSD)^c^	
Yes	38 (20.7)
MINI-assessed Major depressive disorder (MDD)	
Yes	56 (30.3.7)
Either PTSD or MDD (MINI)	
Yes	61 (33.0)

^a^Total *n*s may be less than 185 due to missing data

^b^Refers to valid percentage, excluding missing data

^c^*n* = 184

**Table 3:** Response frequencies for STAR-MH items

	Item	Yes	No
		*n* (%)^a^	*n* (%)^a^
3.	Have you felt very restless, like you can’t keep still?	55 (29.7%)	130 (70.3%)
4.	Have you lost interest in things?	55 (29.7%)	130 (70.3%)
5.	Have you worried about going crazy or ‘losing your mind’?	48 (25.9%)	137 (74.1%)
6.	Have you had a lot of trouble sleeping?	62 (33.5%)	122 (66.5)
7.	Have you felt very fearful?	62 (33.5%)	123 (66.5%)
8.	Have you felt very trapped or caught?	59 (31.9%)	126 (68.1%)
9.	Have you had a lot of pain in your body?	61 (33.0%)	124 (67.0%)
10.	Have you felt very worthless?	54 (29.2%)	131 (70.8%)

*Note*. ^a^Percentages based on *N* = 185

**Fig. 2:**
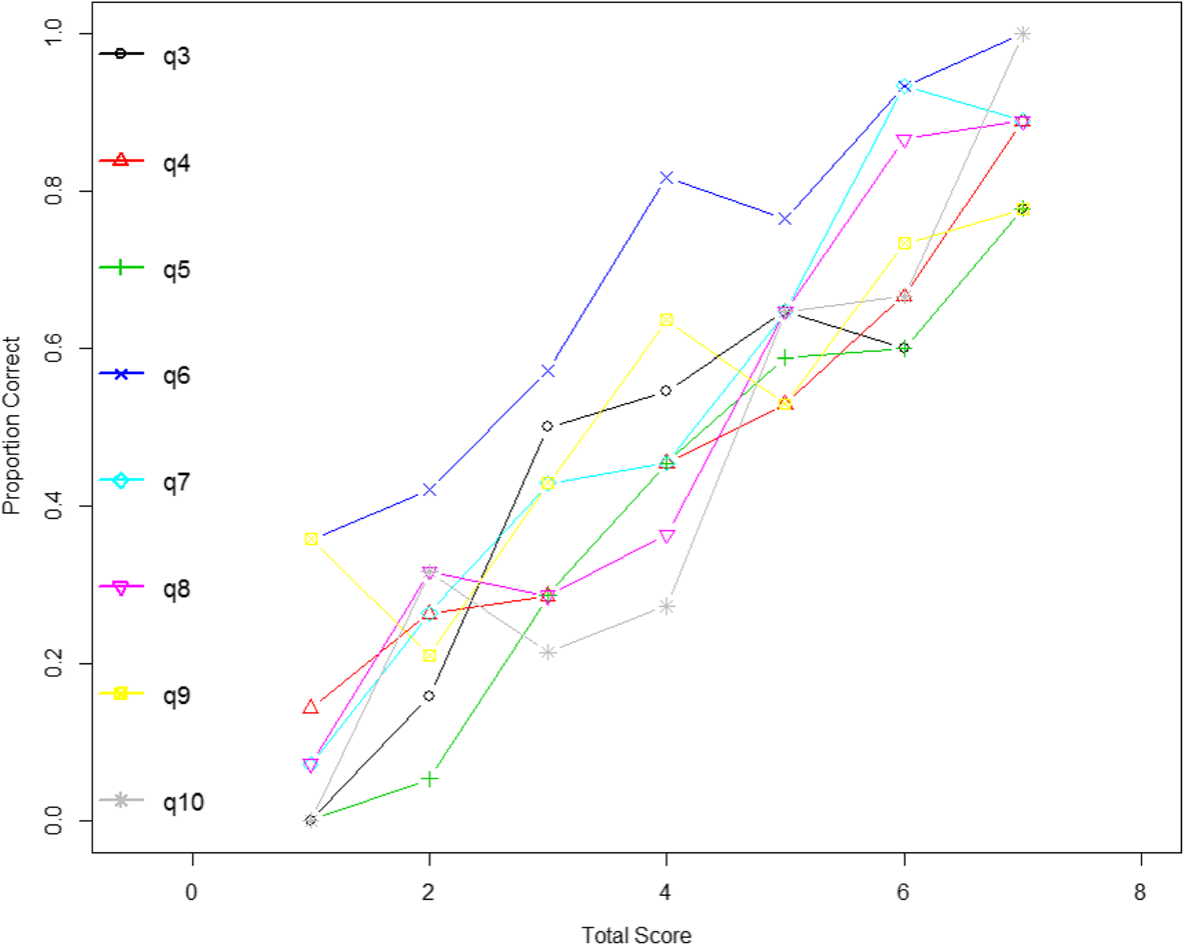
Total score versus proportion of endorsed (correct) responses for items 3–10

The plot of the total score versus the proportion of endorsed responses for each item (see Fig. 2) revealed low variance for item 6 (sleep), demonstrating a 30% chance that individuals would endorse this item even if they did not endorse other items. Furthermore, the probability of endorsing the sleep item increased as respondents endorsed other items (i.e., higher total score). Based on these findings of unacceptably low specificity item 6 was dropped from subsequent analyses.

### Rasch analysis

#### Item fit

Table 4 presents the item fit statistics from the Rasch analysis. All items had an infit statistic between 0.91 and 1.20, and an outfit statistic between 0.85 and 1.27. Similarly, the outfit-*t* statistic ranged from − 1.09 to 1.97, and the infit-*t* statistic ranged from 0.89 to 1.20. The chi-squared test for each item was not statistically significant. This pattern of results indicated that none of the items were misfitting.

**Table 4:** STAR-MH item difficulty estimates, chi-squared test, outfit, and infit statistics, and standardised outfit and infit statistics

Item	Difficulty	*SE*	χ^2^	*df*	*p*	Outfit	Infit	Outfit-*t*	Infit-*t*
Have you felt very restless, like you can’t keep still?	0.06	0.20	98.6	94	1	1.05	1.06	0.41	1.06
Have you lost interest in things?	0.06	0.20	99.4	94	0.269	1.06	1.02	0.47	1.02
Have you worried about going crazy or ‘losing your mind’?	0.40	0.21	80.2	94	1	0.85	0.95	−0.99	0.95
Have you felt very fearful?	−0.27	0.20	81.8	94	1	0.87	0.91	−0.99	0.91
Have you felt very trapped or caught?	− 0.13	0.20	90.5	94	1	0.96	0.97	−0.24	0.97
Have you had a lot of pain in your body?	−0.22	0.20	119.6	94	1	1.27	1.20	1.97	1.20
Have you felt very worthless?	0.11	0.20	80.5	94	1	0.86	0.89	−1.09	0.89

#### Dimensionality and local dependence

The unidimensionality test was not statistically significant (*p* = 0.785), suggesting that the STAR-MH was unidimensional. Ponocny’s “T1” test indicated that there were no locally dependent items (all *p*-values > 0.05).

#### Differential item functioning (DIF)

The analyses indicated that there was a moderate effect of DIF (*R*^2^ = 0.04, *p* = 0.023) for item 4 (*Have you felt very fearful?*) caused by support agency. The ARC group were less likely to endorse this item. No other instances of DIF were detected for sex, age, interpreter use, country of origin, marital status, travel mode, and post-migration detention status.

#### Internal consistency

The PSI for the 7-item STAR-MH scale was 0.75, which indicated good usability at the group levels, but a lack of sensitivity for individual analyses. However, the average inter-item correlation (*r* = 0.46) suggested adequate internal consistency given the small number of items [70].

### ROC analyses

**Fig. 3:**
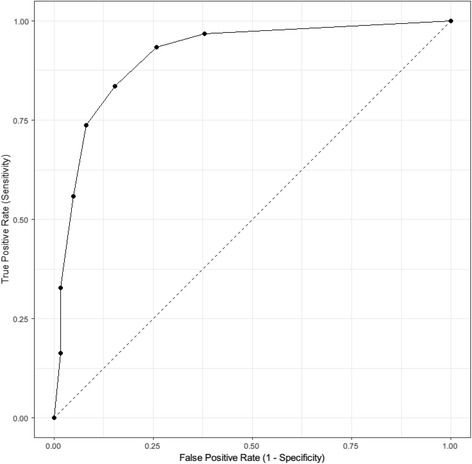
Receiver operating characteristic plot of STAR-MH scores of participants with caseness for PTSD/MDD and those without

Figure 3 presents the ROC plot for participants with caseness for PTSD/MDD compared to those without. The AUC for this analysis was 0.912 (95% CI = 0.868–0.956). Using bootstrap validation, the optimism-corrected AUC was 0.911, which represents the predictive ability of the model in future forced migrants.

Table 5 shows that a STAR-MH cut-off score of 3 produced the best balance of sensitivity and specificity based on Youden’s *J*. While a cut-off score of ≥2 for the 7-item scale gave a lower positive predictive value than a score of ≥3, it provided higher negative predictive value, which was a desirable requisite for the screening tool. A cut-off score of three rather than two resulted in a greater PLR, with moderate utility (~ + 20–30% change in probability) whilst the NLR was in the moderate to high range (~ − 30– 45% change in probability) for both cut points [76]. Similarly, the overall diagnostic accuracy was above 80% for both.

**Table 5:** Receiver operating curve analysis, likelihood ratios and predictive values of the STAR-MH

Score	Sensitivity % (95% CI)	Specificity % (95% CI)	PLR (95% CI)	NLR (95% CI)	PPV % (95% CI)	NPV % (95% CI)	Efficiency (95% CI)	Youden’s *J*
1	96.7% (88.7, 99.6)	63.1% (54.2, 71.4)	2.6 (2.1, 3.3)	0.1 (0.0, 0.2)	55.6% (45.7, 65.1)	97.6% (91.7, 99.7)	74.0% (67.1, 80)	1.60
2	93.4% (84.1, 98.2)	74.6% (66.2, 81.8)	3.7 (2.7, 5)	0.1 (0.0, 0.2)	63.7% (53, 73.6)	96% (90.2, 98.9)	80.7% (74.4, 86.1)	1.68
**3**	83.6% (71.9, 91.8)	84.6% (77.2, 90.3)	5.5 (3.6, 8.3)	0.2 (0.1, 0.3)	72.2% (60.4, 82.1)	91.7% (85.2, 95.9)	84.4% (78.5, 89.2)	1.69
4	73.8% (60.9, 84.2)	91.5% (85.4, 95.7)	8.8 (4.9, 15.7)	0.3 (0.2, 0.4)	80.7% (68.1, 90)	88.1% (81.5, 93.1)	85.9% (80.2, 90.5)	1.66
5	55.7% (42.4, 68.5)	95.4% (90.2, 98.3)	12.2 (5.4, 27.5)	0.5 (0.3, 0.6)	85.4% (70.8, 94.4)	82.1% (75.1, 87.9)	82.8% 76.7, 87.9)	1.52
6	32.8% (21.3, 46)	98.5% (94.6, 99.8)	21 (5.1, 86.9)	0.7 (0.6, 0.8)	90.9% (70.8, 98.9)	75.3% (68.1, 81.6)	77.1% (70.5, 82.8)	1.31
7	16.4% (8.2, 28.1)	98.5% (94.6, 99.8)	10.5 (2.4, 46.4)	0.9 (0.8, 1.0)	83.3% (51.6, 97.9)	71.1% (63.9, 77.6)	71.9% (64.9, 78.1)	1.15

*Note. PLR* positive likelihood ratio, *NLR* negative likelihood ratio, *PPV* positive predictive value, and *NPV* negative predictive value

## Discussion

This paper presents the psychometric properties and utility of a screening tool for use by non-mental health practitioners to screen for mental disorders in asylum-seekers and new refugees (ASR). Our aim was to develop a brief, highly sensitive and easily administrable tool which would alert non-mental health workers of the need to refer a positively screened individual for a mental health evaluation.

The resultant STAR-MH is a psychometrically robust 9-item screening tool comprising two ‘immediate screen-in’ items and a 7-item scale with a cut-off score of ≥2. The administration time was six minutes, with or without an interpreter, whilst noting in routine use that a positive response to either immediate screen-in item (Items 1 or 2) would obviate the need to continue the screen. Therefore, based on our findings, those who screened positive (22.7%; *n* = 42) to one of the first two items would have been effectively screened in less than 3 min.

Given the small number of items comprising the STAR-MH, internal consistency was sound, and the Rasch analyses confirmed the unidimensionality of the 7-item scale and goodness-of-fit of all items. It performed well in the latent construct of psychological distress according to the differential item functioning (DIF) across all subgroups apart from one pilot site for item 7 (*Have you felt very fearful*?). A possible explanation may lie in differing ecological influences between the ASRC and MHC that were not accounted for by the pilot. Further post hoc analyses would need to be conducted to elucidate this, particularly the relationship between fear and psychological distress for this population. However, a putative explanation may lie in the high degree of psychosocial support received by ASRC participants. The protective role of social-emotional support in the mental health of forced migrant populations has been well-established [14, 77].

The ROC curve indicated the STAR-MH to be performing in the excellent range, with a diagnostic accuracy of between 81 and 84%, depending on the cut-off score. The empirically derived cut point suggested an optimum cut-off score of ≥3, however privileging sensitivity to minimise screening out ‘true’ cases of PTSD or MDD indicated a cut-off score of ≥2. It is anticipated that field testing in a larger ASR sample population will clarify the optimal cut-off score, however, ≥ 2 is most consistent with the aim of optimising sensitivity. In depth testing within specific language and ethnic groups would be justified to confirm the validity of these findings. However, the findings from the bootstrap validation analyses suggest that the diagnostic accuracy of the STAR-MH will not likely be diminished when used with other forced migrant populations.

Notwithstanding the inadequacies of the Western diagnostic lens, PTSD is the most ‘robust’ epidemiological diagnostic construct that we have for assessing and treating trauma-related symptomatology. However, it is important to note that diagnostic criteria for disorders such as PTSD and depression are limited in their ability to predict distress and impairment across diverse linguistic and cultural groups. Hence, the items resulting in the final version of the STAR-MH were derived inductively, exploiting measures from scales that have been adapted for use in a range of cultural groups to maximise cultural sensitivity. It is therefore reasoned that the items comprising the STAR-MH reflect trauma manifestations of forced migrant populations rather than corresponding directly to the Western construct of symptoms.

Study limitations included the relatively small sample, consistent with sampling difficulties characteristic of asylum-seeker populations in general [78]. Despite this, the prevalence of PTSD and/or MDD found in this population (32%) was consistent with rates of these disorders found in other ASR populations internationally [10]. Nonetheless, field testing will need to be undertaken to confirm the validity and reliability of the STAR-MH in larger ASR sample populations. Whilst we endeavoured to recruit both a representative and heterogeneous group of administrators and ASR participants (i.e., culturally and linguistically diverse sample), until field studies have been undertaken, the external validity of the tool must be interpreted with caution.

Although administrators were instructed to read the STAR-MH items faithfully and neutrally, no systematic administrator observation was undertaken. In situ translation with different interpreters also presents an increased risk to the fidelity of administration. This raises the critical issue of how items were verbally translated by interpreters into culturally valid idioms. However, the STAR-MH is an amalgam of items from gold standard measures of PTSD and MDD symptomatology in ASR populations [35, 79]. Furthermore, leaders from several ethnic communities were consulted about the cultural sensitivity and utility of the STAR-MH and endorsed it for use in their respective communities.

Whilst the brevity and simplicity of the tool means that administrator training is not necessary (i.e., the worker need only follow the instructions on the form itself), the STAR-MH is not designed for self-administration or for lay administration but rather for a worker in the field. This is to ensure that a referral processes can be instigated in the event of a positive screen result and/or an abreaction during the screening process, although none of the latter were noted in this study.

The STAR-MH differentiates itself from related tools in its screening breadth and predictive validity. Hence, in comparison to other widely used screening tools (e.g., K10 and PTSD-8), the STAR-MH screens for both PTSD and MDD. The high comorbidity of PTSD and depression in forced migrant populations [80–82], necessitates a tool that can efficiently screen for both disorders. Unlike the K10 and RHS-15, the STAR-MH has clinical predictive validity, having been validated against a diagnostic instrument in both primary health and community settings. In contrast, relatively high rates of misclassified true cases and non-cases in studies that utilised the K10 in culturally diverse populations have raised questions about its suitability for non-Western groups [17].

## Conclusion

The STAR-MH is a simple, sensitive screening tool to facilitate mental health referrals for asylum-seekers and new refugees at the agency of first presentation. The pilot of a 9-item version has demonstrated promising results ahead of field testing to ascertain its external validity in community-dwelling asylum-seeker and new refugee populations in industrialised host nations.

## Additional files

Additional file 1: **Table S1.** Demographic and clinical variables of participants (*N* = 192). Demographic and clinical variables of participants for the total sample, including cases with missing variables. (PDF 152 kb)

Additional file 2: **Table S2.** Response frequencies for STAR-MH items (*N* = 192). Response frequencies for STAR-MH items 3–10 for total sample, including cases with missing variables. (PDF 243 kb)

## Abbreviations

ASR: Asylum seekers and refugees asylum-seekers
ASRC: Asylum Seeker Resource Centre
AUC: Area under the curve
CI: Confidence intervals
DIF: Differential Item Functioning
GP: General Practitioner
HSCL-25: Hopkins Symptom Checklist-25
HTQ: Harvard Trauma Questionnaire
M.I.N.I.: Mini International Neuropsychiatric Interview
MDD: Major depressive disorder
MHC: Monash Community Health
NLR: Negative likelihood ratio
NPV: Negative predictive value
PERI-D: Psychiatric Epidemiology Research Interview–Demoralisation Scale
PLR: Positive likelihood ratio
PMLDC: Post-migration Living Difficulties Checklist
PPV: Positive predictive value
PSI: The Person Separation Index
PTSD: Post-traumatic stress disorder
ROC: Receiver operating characteristic
SN: Sensitivity
SP: Specificity
STAR-MH: Screening tool for asylum seeker and refugee mental health
WHO: World Health Organisation
