# Supplementary material for: Development and validation of a mental health screening tool for asylum-seekers and refugees: the STAR-MH
Source: BMC Psychiatry. 2018 Mar 16;18:69. doi: 10.1186/s12888-018-1660-8 (PMC5857116; doi:10.1186/s12888-018-1660-8)
Supplement: Supplementary file 1 — Table S1. Demographic and clinical variables of participants (N = 192). Demographic and clinical variables of participants for the total sample, including cases with missing variables. (PDF 152 kb) [file 12888_2018_1660_MOESM1_ESM.pdf]

Table S1

Demographic and clinical variables of participants (*N*=192)

	N [a] *(%)* [b]
**Gender**	
Male	134 (69.8)
**Age group**	
18-24	24 (12.5)
25-34	79 (41.1)
35-44	49 (25.5)
45-54	28 (14.6)
55+	12 (6.3)
**Marital status**	
Partnered	117 (60.9)
**Mode of Arrival**	
Irregular maritime arrival	104 (54.2)
**Continent of Origin** (UN geoscheme)	
Africa	
East Africa	17 (9.1)
North Africa	10 (5.3)
West Africa	7 (3.7)
Asia	
South Asia	97 (51.9)
South-East Asia	39 (20.9)
West Asia	8 (4.3)
Other	9 (4.7)
**Interpreter required**	
Yes (screening)	118 (61.5)
Yes (interview)	125 (65.1)
**Pre-migration camp/detention**	
Yes	28 (14.8)
**Post-migration Immigration Detention**	
Yes	105 (54.7)
**Years in Australia**	
< 1 year	36 (19.0)
1-2 years	34 (18.0)
2-3 years	79 (41.8)
> 3 years	40 (21.2)
**Residency status**	
Temporary Visa (Asylum-seeker)	187 (97.4)
Permanent Residency (Refugee)	5 (2.6)
**Mental health diagnosis (Australia)**	
Yes	8 (4.4)
**Post-traumatic stress disorder (PTSD)** [c]	

Yes	38 (19.9)
**Major depressive disorder (MDD)**	
Yes	57 (29.7)
**Either PTSD or MDD**	
Yes	62 (32.3)

[a] Total *n*s may be less than 192 due to missing data
[b] Refers to valid percentage, excluding missing data.
[c] *n* = 191.
