# Supplementary material for: Development and validation of a mental health screening tool for asylum-seekers and refugees: the STAR-MH
Source: BMC Psychiatry. 2018 Mar 16;18:69. doi: 10.1186/s12888-018-1660-8 (PMC5857116; doi:10.1186/s12888-018-1660-8)
Supplement: Supplementary file 2 — Table S2. Response frequencies for STAR-MH items (N = 192). Response frequencies for STAR-MH items 3–10 for total sample, including cases with missing variables. (PDF 243 kb) [file 12888_2018_1660_MOESM2_ESM.pdf]

Table S2

Response frequencies for STAR-MH items [a, b]

Item	Missing *n* (%)	Yes *n* (%)[a]	No *n* (%)[a]
3. Have you felt very restless, like you can't keep still?	0 (0.0%)	56 (29.2%)	136 (70.8%)
4. Have you lost interest in things?	0 (0.0%)	56 (29.2%)	136 (70.8%)
5. Have you worried about going crazy or 'losing your mind'?	0 (0.0%)	49 (25.5%)	143 (74.5%)
6. Have you had a lot of trouble sleeping?	0 (0.0%)	80 (41.7%)	112 (58.3%)
7. Have you felt very fearful?	0 (0.0%)	63 (32.8%)	129 (67.2%)
8. Have you felt very trapped or caught?	0 (0.0%)	60 (31.2%)	132 (68.8%)
9. Have you had a lot of pain in your body?	2 (1.0%)	63 (32.8%)	129 (67.2%)
10. Have you felt very worthless?	5 (2.6%)	56 (29.2%)	136 (70.8%)

[a] 8-item subscale

[b] Percentages based on $N = 192$
